# High mortality due to accidental salinomycin intoxication in sheep

**DOI:** 10.2478/intox-2014-0024

**Published:** 2014-12-30

**Authors:** Javad Ashrafihelan, Hamed Eisapour, Amir Mehdi Erfani, Amir Ali Kalantary, Jamileh Salar Amoli, Morteza Mozafari

**Affiliations:** 1 Department of Pathobiology, Faculty of Veterinary Medicine, University of Tabriz, Tabriz, Iran; 2 Veterinary Organization, Zanjan, Iran; 3 Toxicology Research Center, Faculty of Veterinary Medicine, University of Tehran, Tehran, Iran; 4 Faculty of Veterinary Medicine, University of Tabriz, Tabriz, Iran

**Keywords:** salinomycin, sheep, acute toxicosis, cardiomyolysis, acute tubular necrosis, Iran

## Abstract

In February 2012, 100% mortality was reported in a herd with 79 local sheep that were kept around of Abhar, Northwest of Iran. The ration for adult sheep was daily mixed (40 kg straw, 25 kg wheat and 2 kg Vit-C premix) and accidentally 1 500 g of salinomycin (Salinomycin 12% Premix; Aras Bazar Laboratories, Iran) had been added to the ration (22388 mg/kg = 22388 ppm) and overnight was fed to herd. At the morning, 78 sheep were founded dead and one of them showed convulsive seizures. Postmortem examination revealed pulmonary congestion and edema, hemorrhages in abomasum, large pale kidney and white streak lines in myocardium. Main histopathologic lesions were extensive subepicardial and intercardiomyofibers hemorrhages, extensive cardiomyolysis and myocarditis in heart, severe hyperemia and extensive acute tubular necrosis (ATN) in kidneys and focal necrosis and retention of bile cholangitis in the liver. In this study, on the basis of the history, observation of the ionophore remnant in the ration, clinical signs, gross and histopathological findings, acute salinomycin intoxication is definitely diagnosed.

## Introduction

Salinomycin, monensin, lasalocid, narasin, and maduramicin are ionophores and carboxylic polyether antibiotics with antimicrobial and anticoccidial properties (Novilla, 1992; Radostits *et al*., 2007). Ionophores are used as anticoccidial drugs for poultry. Dairy farmers use ionophores to increase milk production, prevention of bovine acute pulmonary edema and emphysema (BAPEE), rumenal lactic acidosis and decreased incidence of bloat and amelioration of ketosis in lactating cattle (Russell & Houlihan, 2003; Novilla, 1992; Radostits *et al*., 2007; Gupta, 2007). However, because of a narrow safety margin and careless use, ionophores have been associated with major losses (Radostits *et al*., 2007). Toxicity of these compounds varies with the particular ionophore compound and the species and age of animals (Wilson, 1980; Hanson *et al*., 1981; Galitzer *et al*., 1986).

Salinomycin produced by the fermentation of fungal Streptomyces species which has activity against some gram-positive bacteria, coccidia, neospora, and toxoplasma (McKellar & Lawrence, 1996).

Salinomycin is used in chickens for fattening with a maximum content of the active ingredient in feed of 70 mg/kg and a withdrawal period of one day, for chickens reared for laying (up to 12 weeks of age) with a maximum content of 50 mg/kg and no withdrawal period, and for rabbits for fattening with a maximum concentration in feed of 25 mg/kg and a withdrawal period of five days (European Food Safety Authority, 2008).

Also, as other ionophores, toxic dose of salinomycin varies and depends on the species and age of animal in affected cases or use of it in non-target animals (Plumlee *et al*., 1995; Wilson 1980; Hanson *et al*., 1981; Galitzer *et al*., 1986). But dosage and withdrawal times in ruminants are not presented in the literatures.

Salinomycin intoxication has been described in turkey (Potter *et al*., 1986; Griffiths *et al*., 1989; Andreasen & Schleifer, 1995; Assen, 2006), horse (Van Amstel & Guthrie, 1985; Rollinson *et al*., 1987; Aleman *et al*., 2007), pig (Miller *et al*., 1986; Kavanagh & Sparrow, 1990), cat (Van Der Linde-Sipman *et al*., 1999) and cattle (Huyben *et al*., 2001) throughout of the world. An accidental toxicosis in calves (Omidi *et al*., 2010) and an experimental toxicosis in sheep were previously reported from Iran (Tafti *et al*., 2008). This communication describes accidental salinomycin toxicosis with high mortality in sheep around of Abhar, Zanjan province, Northwest of Iran.

### Materials and methods

#### Case description

In February 2012, a high mortality was reported in a herd with 79 local sheep that were kept around of Abhar, Zanjan province, Northwest of Iran with some local cows, goat and domestic foals and donkey. Investigation of stockyard and rations was revealed the ration for adult sheep was daily mixed and contained 40 kg straw, 25 kg wheat and 2 kg Vit-C premix. The animals were fed on pasture in the morning and the mixed ration in the evening. One thousand five hundred grams (1 500 g) of salinomycin (Salinomycin 12% Premix; Aras Bazar Laboratories, Iran) had blunderingly been mixed with ration (22388 mg/kg = 22388 ppm) and overnight was fed to herd. Seventy eight sheep were founded dead at the morning and one of them was moribund and showed frothing at the mouth, opisthotonos and convulsive seizures. The remnants of the ration mixed with salinomycin were noticed in the manger and salinomycin particles as whitish powder was grossly detected.

#### Histopathological examination

Tissue samples of heart, liver, and kidneys were taken for histopathological examination. Sheep carcasses were all buried underground to minimize health risks for humans and carnivores. Samples were fixed in 10% neutral buffered formalin, processed with standard histological method and stained with hematoxylin and eosin.

## Results

At necropsy, the main gross findings were pulmonary congestion and edema, accumulation of whitish frothy content in respiratory airways (shock lung), hemorrhagic foci in abomasum, intestinal congestion, gall bladder distention, congestion, swelling and nutmeg appearances in liver, large pale kidney and severe congestion and white streak lines in myocardium. Other tissues were congested.

**Figure 1:**
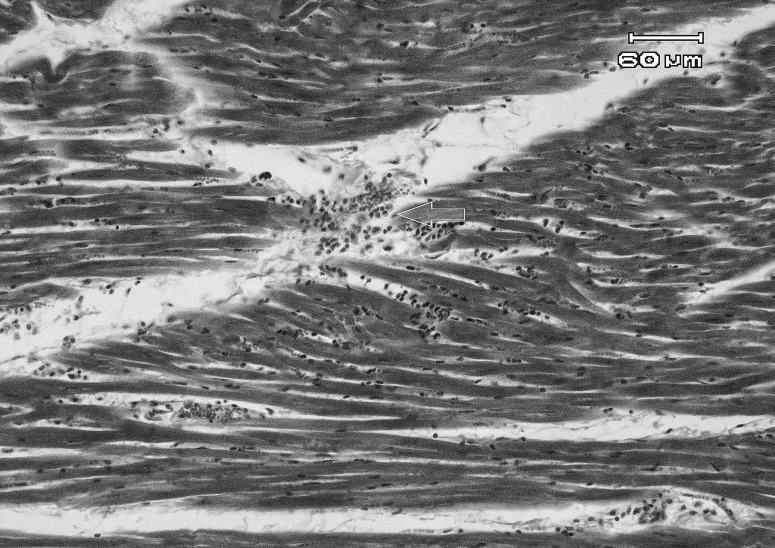
Microscopic view of heart in a sheep affected with acute salinomycin intoxication: focal hyaline degeneration, cardiomyolysis and focal myocarditis (arrow) are noticed (H&E, 200×).

**Figure 2:**
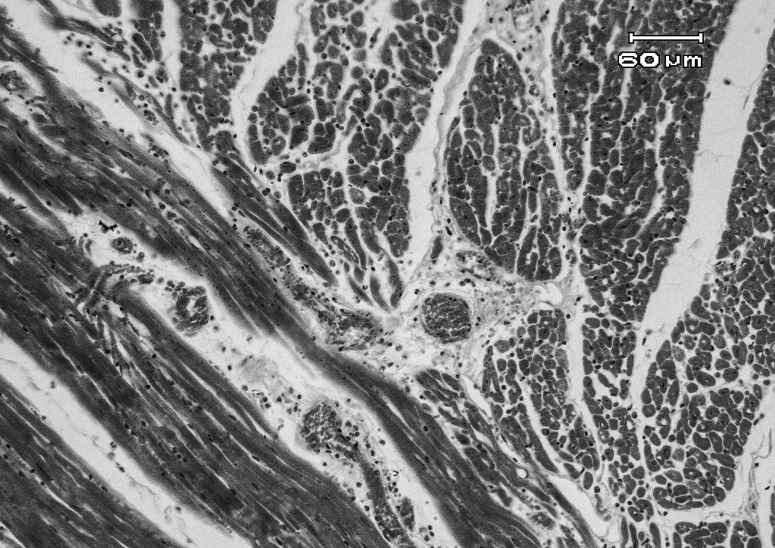
Microscopic view of heart in a sheep affected with acute salinomycin intoxication: congestion, edema, and infiltration of inflammatory cells are seen (H&E, 200×).

**Figure 3:**
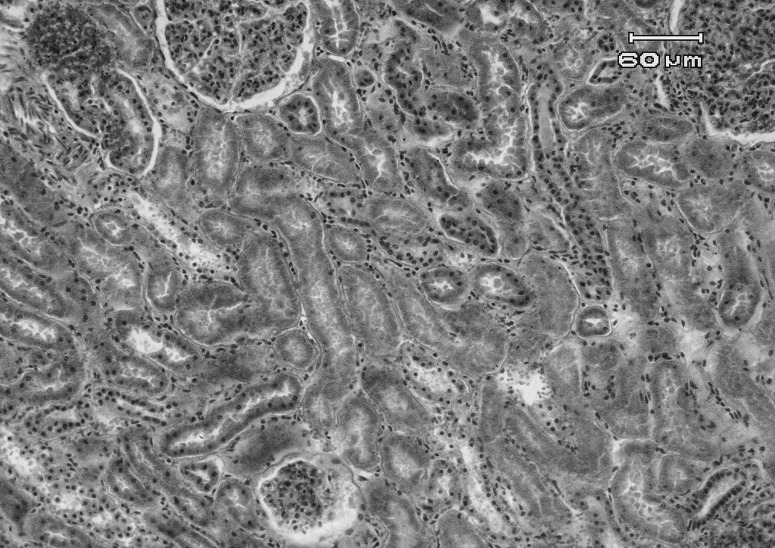
Microscopic view of kidney in a sheep affected with acute salinomycin intoxication: extensive coagulative necrosis of renal tubules (nephrotic ATN) is observed (H&E, 200×).

**Figure 4:**
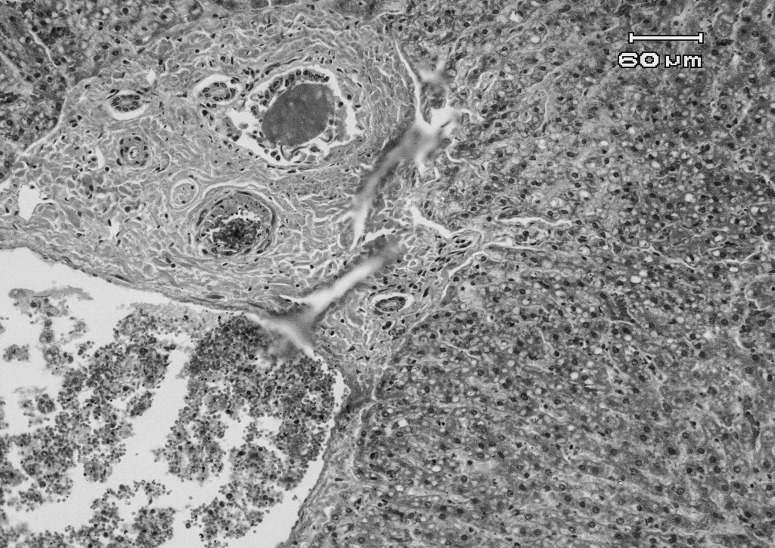
Microscopic view of liver in a sheep affected with acute salinomycin intoxication: congestion, mild fatty change of hepatocytes and periportal fibrosis are noticed (H&E, 200×).

Histopathological examination showed extensive subepicardial hemorrhages, edema and hemorrhages between cardiomyocytes bundles, extensive myocardial degeneration and necrosis (cardiomyolysis), with infiltration of mononuclear and PMN inflammatory cells in myocardium (Figures 1 & 2). Also a number of small to large cysts of sarcocystis in the myocardial cells were found. In the kidney, severe hyperemia, extensive cell swelling and multifocal nephrotoxic acute tubular necrosis (ATN) of proximal convoluted tubules were observed (Figure 3). Severe hyperemia, mild fatty change, focal necrosis, retention of bile in bile canaliculi, cholangitis and periportal fibrosis were noticed in liver (Figure 4). Other tissues were severely congested and edematous.

## Discussion

Presently, seven carboxylic ionophores (monensin, lasalocid, salinomycin, narasin, maduramicin, laidlomycin and semduramicin) are approved for the control of coccidiosis and promotion of growth and feed efficiency in several animals of economic importance. Carboxylic ionophores have played significant roles in livestock and poultry production systems throughout the world (Gupta, 2007). Generally, marketed ionophore products have been found to be safe and effective in the target species provided the approved dosage ranges (Gupta, 2007). However, excessive ionophore feed concentrations have resulted in the ionophore toxic syndrome (Gupta, 2007; Novilla, 1992). Feed-mixing errors have caused most toxicity problems in animals for which ionophore use has been approved.

Ionophores toxicity varies considerably among species, with horses being the most sensitive. Toxicity may occur after accidental access to medicated feed, errors in feed mixing, deliberate feeding of a ration formulated for a less sensitive species, and or concurrent use of some products with known interactions such as tiamulin, oleandomycin, chloramphenicol, erythromycin, and sulfonamides (Gupta, 2007; Roder & McCoy, 1999).

All ionophores facilitate transmembrane ion fluxes and dissipation of ion gradients, which are exaggerated at toxic levels. Cells respond to the metabolic insult by expending energy to maintain homeostasis. When homeostatic mechanisms are exceeded, toxicity ensues from excessive influxes of cations leading to degeneration and necrosis of cardiac and skeletal muscle cells. Salinomycin is a monovalent ionophore that has higher affinity for K^+^ than Na^+^. Binding to K^+^ can cause loss of intracellular potassium, which results in inhibition of ATP hydrolysis in the mitochondria with subsequent decreased cell energy production and death (Aleman *et al*., 2007).

Inotropic and chronotropic properties of salinomycin have been described (Fahim *et al*., 1986). Sudden death in weeks or even months following ingestion of ionophores has been reported (Schweitzer *et al*., 1984; McKellar & Lawrence, 1996). Degenerative myopathy and myocardiopathy are the main injuries reported in the affected animals (Barros, 1998), because ionophores form complexes with cations and mediate their transport across the cell membrane in response to diffusion gradient, mitochondrial failure and depletion of cellular adenosine triphosphate (ATP) may occur, therefore highly energetic tissues of the body are primarily affected (Gupta, 2007).

Sheep and goats have similar clinical signs like anorexia, diarrhea and ataxia but affected lambs frequently exhibit labored breathing, frothing at the mouth, and kicking at the abdomen (Gupta, 2007; Agaoglu *et al*., 2002).

Necropsy findings in animals with ionophore toxicosis include hemorrhages and pale areas in the heart and limb muscles, pulmonary edema, hydrothorax, ascites, and inflammation of the stomach and intestines (Gupta, 2007; Galitzer *et al*., 1986; Salles *et al*., 1994). But Animals that die soon after exposure might don't have any lesions.

Target organs damaged by toxic doses ionophores were identified to include the heart and skeletal muscles in all species studied (Gupta, 2007; Novilla & Folkerts, 1986). In addition, neurotoxic effects have been reported for lasalocid (Shlosberg *et al*., 1985; Safran *et al*., 1993), narasin (Novilla *et al*., 1994), and salinomycin (Van der Linde-Sipman *et al*., 1999). Generally, no significant lesions are seen by light microscopy in animals that die immediately, and animals that die after an acute course may have only a few scattered degenerated fibers in the heart and highly active muscles. The most important change is a toxic myopathy characterized by focal areas of degeneration, necrosis, and repair in cardiac and skeletal muscles with a variable inflammatory component (Gupta, 2007; Novilla & Folkerts, 1986). Therefore in present study, heart, skeletal muscles, liver, lungs and especially kidneys were sampled for histopathological studies.

To author's knowledge, this is the first report of accidental salinomycin intoxication in sheep with 100% mortality rate from Iran. In this study, postmortem examination revealed pulmonary congestion and edema, abomasal hemorrhages, large pale kidney and white streak lines in myocardium. Main histopathologic lesions were extensive cardiac hemorrhages, extensive cardiomyolysis, myocarditis, acute tubular necrosis (ATN) and focal necrosis and retention of bile in the liver. The clinical and histopathological findings were relatively consistent with previous reports of salinomycin and other ionophores intoxication in Iran and other countries.

In the present report, 79 adult sheep exposed to 22388 ppm salinomycin, showed acute intoxication and high mortality. Previously intoxication and mortality (67%), in 16-week old calves, exposed to a 70 000 ppm salinomycin has been reported from Iran (Omidi *et al*., 2010). On the basis of previously mentioned points about salinomycin indication in poultry and rabbit, if normal dosage of salinomycin is assumed 50 mg/kg of ration, it seems the affected animals were exposed with approximately 447 times in comparison of the normal daily use in the farm. Salinomycin particles as whitish powder were grossly detected in the remnants of the mixed ration in the manger. Seventy eight sheep were founded dead and one of them was moribund and showed convulsive seizures. Mortality was 100%. The other animals hadn't received that ration and were health.

Although, clinical signs and pathologic lesions are not pathognomonic for definitive diagnosis of ionophores intoxications and requires toxicological analysis of the feed, in this study on the basis of the history, observation of the ionophore remnant in the ration, clinical signs, gross and histopathological findings, acute salinomycin intoxication is definitely diagnosed.
